# Contrasting Effects of Grass - Endophyte Chemotypes on a Tri-Trophic Cascade

**DOI:** 10.1007/s10886-020-01163-9

**Published:** 2020-03-03

**Authors:** Benjamin Fuchs, Eric Kuhnert, Jochen Krauss

**Affiliations:** 1 Biodiversity Unit, University of Turku, 20014 Turku, Finland; 2 Institute for Organic Chemistry, BMWZ, Leibniz Universität Hannover, Schneiderberg 38, 30167 Hannover, Germany; 3 Department of Animal Ecology and Tropical Biology, Biocentre, University of Würzburg, Am Hubland, 97074 Würzburg, Germany

**Keywords:** Pest-control, Trophic interactions, Endophytes, Plant defense, Biocontrol, Plant-associated microorganisms, Phytobiome

## Abstract

Systemic grass-endophytes of the genus *Epichloë* symbiotically infect the above-ground plant parts of many grass species, where they produce alkaloids in a grass- and endophyte-specific manner that are toxic or deterrent to herbivores. An increasing number of studies show cascading negative effects of endophyte-derived alkaloids that extend to higher trophic levels, harming beneficial insects, including those that control aphid populations. Lacewings are one of the major biological aphid controls, and are especially resistant to insecticides and pollutants, but their susceptibility to endophyte infection in the food chain has never been studied. Our study found variability in aphid population growth depending on the endophyte-grass chemotype, where aphid population growth was lowest on chemotypes known for producing high amounts of loline alkaloids. We also showed that larval and pupal development and mortality of the Common Green Lacewing (*Chrysoperla carnea*) was, in a non-choice experiment, not affected by endophyte infection in the food chain. This is a first indication that lacewings might be resistant to endophyte-derived alkaloids and could be robust biocontrol agents when applied together with endophyte-infected grass, possibly replacing chemical pesticides.

**Electronic supplementary material:**

The online version of this article (10.1007/s10886-020-01163-9) contains supplementary material, which is available to authorized users.

## Introduction

Pest control in agricultural systems has relied for many decades on the application of chemical pesticides to control plant damage caused by insect herbivores. Recent approaches for the prevention of pest infestation in agricultural systems have moved towards sustainable, environmentally friendly methods based on natural modes of pest control (van Lenteren et al. 2018). Sustainable insect pest control strategies involve natural enemies (top-down control) of insects, such as predatory and parasitoid insects (Thurman et al. 2017), but further rely on resistant plant cultivars (Stenberg 2017). Plant-associated microorganisms are increasingly being discovered and applied as either entomopathogenic agents or as plant mutualists, boosting defenses against insect pests by increased production of deterrent or toxic metabolites (Gange et al. 2019; Kauppinen et al. 2016; Vega 2018).

Asexual representatives of systemic fungal endophytes (genus *Epichloë*) infecting cool-season grasses can be plant mutualistic, improving plant traits such as drought tolerance and herbivore resistance (Saikkonen et al. 1998, 2013). The latter trait results from production of several alkaloids such as lolitrem B and ergopeptine alkaloids (e.g., ergovaline), which result in severe diseases in livestock, and peramine, lolines, ergopeptine alkaloids and epoxy-janthitrems, which are toxic and/or deterrent to insect pests (Bush et al. 1997; Leuchtmann et al. 2000; Panaccione et al. 2014). Each grass-endophyte symbiosis produces a specific alkaloid profile in which the amount produced can be affected by biotic and abiotic conditions, but aphid herbivory has not been shown to affect alkaloid concentrations in a common garden study (Bultman et al. 2004; Fuchs et al. 2017b, 2017c; Helander et al. 2016).

Pastures and meadows are often dominated by single grass species which comprise the food supply to livestock (Plantureux et al. 2005). Tall fescue (*Festuca arundinacea*) and perennial ryegrass (*Lolium perenne*) are distributed worldwide and are agriculturally important grass species (Cougnon et al. 2014; Hoveland 2009). Both species can be found in association with different strains of endophytic fungi. Each plant-endophyte symbiosis produces strain-specific alkaloids leading to different chemotypes (Schardl et al. 2013; Takach et al. 2012; Takach and Young 2014). The common strain (CS) of the asexually reproducing endophytic fungus *Epichloë coenophiala* is symbiotic with *Festuca arundinaceae* and produces several pyrrolizidine alkaloids (N-formylloline, N-acetylloline and N-acetylnorloline), the pyrrolopyrazine alkaloid peramine, and the vertebrate-toxic ergot alkaloid ergovaline (Siegel et al. 1990; Ball and Tapper 1999). The Moroccan strain (licensed by AG Research NZ under the code AR 542) of this endophyte produces alkaloids only effective against insects (N-acetylnorloline and peramine) and is consequently preferably used for pest control on pastures (Ball and Tapper 1999). The common toxic strain of *Epichloë festucae* var*. lolii* is symbiotic with *Lolium perenne* and produces peramine as the main insect-deterring alkaloid, and also the indole diterpene alkaloid lolitrem B and the ergot alkaloid ergovaline, but none of the pyrrolizidine alkaloids (Siegel et al. 1990; Siegel and Bush 1996).

Aphids are a major pest in agricultural systems, directly damaging the plants by piercing-sucking herbivory, and severely harming pastures and crops by transferring disease viruses (Ng and Perry 2004). Grass-endophyte infection can decrease both adult life span and fecundity of certain aphids but these results are often limited to single grass-endophyte associations (e.g. Bastías et al. 2017; Bieri et al. 2009; Meister et al. 2006).

Anti-insect effects of endophytes have been shown for herbivores and higher trophic levels, reducing the fitness of both aphid predators and primary and secondary parasitoids (Bultman et al. 2012; de Sassi et al. 2006; Härri et al. 2008a, 2008b). The alkaloids peramine and lolitrem B from *L. perenne* infected with *E. festucae* var*. lolii* cascade-up the food chain and are probably responsible for fitness reduction of these important predators (Fuchs et al. 2013). To the best of our knowledge, there have been no reports of alkaloids produced by the symbiosis between *E. coenophiala* and *F. arundinaceae* cascading through the food chain, but negative effects on parasitoids resulted from feeding lolines to their lepidopteran host (Bultman et al. 1997). Some mostly specialized herbivorous insects can sequester alkaloids for their own defense (Trigo 2011), but this remains to be elucidated for endophyte-derived alkaloids.

Larvae of the Common Green Lacewing (*Chrysoperla carnea*) are one of the main commercially available aphid-pest control agents, but their susceptibility to endophyte-derived alkaloids is unknown. These insects are known to be resistant to certain pesticides and consequently serve as reliable aphid control in contaminated areas (Pree et al. 1989).

In this study, we compared the effects of three different grass-endophyte strains on aphid population growth. Further, we tested how lacewing larval development is affected when larvae are fed exclusively on aphids from *Epichloë*-infected host grass.

## Methods and Materials

### Studied Material

We analyzed the performance of aphids on five different plant treatments, using three different grass-endophyte strains (*L. perenne* seeds provided by AgResearch NZ, *F. arundinaceae* seeds provided by University of Georgia). Each strain expresses its own unique alkaloid chemotype, which is mainly responsible for its anti-herbivore effects (Saikkonen et al. 2016):

i. *Festuca arundinaceae* symbiotic with the endophytic fungus *Epichloë coenophialia* (common strain) (FE+) producing loline alkaloids (N-formylloline, N-acetylloline und N-acetylnorloline), peramine and ergovaline (Ball and Tapper 1999; Siegel et al. 1990);
ii. *F. arundinaceae* symbiotic with the Moroccan strain of *E. coenophiala* (FEM) producing the alkaloids N-acetylnorloline and peramine (Ball and Tapper 1999);
iii. *F. arundinaceae* not infected with an *Epichloë* endophytic fungi (FE-);
iv. *Lolium perenne* symbiotic with *Epichloë festucae* var. *lolii* (common toxic strain) producing the alkaloids peramine, lolitrem B and ergovaline (LE+) (Siegel et al. 1990; Siegel and Bush 1996);
v. *L. perenne* not infected with an *Epichloë* endophytic fungi (LE-).

For each treatment, 12 pots (13 × 12 × 12 cm) were set up in March, and individually filled with soil (Einheitserde classic) and 200 evenly distributed seeds. Pots were randomly grouped and positioned in a greenhouse with 11 hr of day length exposure in March, increasing to 15 hr during May without additional light or temperature treatment, but with a daily water supply (Fig. S1). After 12 weeks of growth (May), seeds had developed between five and eight tillers with fully developed leaves. Plants did not produce inflorescences at this stage.

*Epichloë* infection of each pot was verified by microscopy following staining one leaf with aniline blue at the end of the experiment. All *Epichloë*-infected plants contained characteristic hyphae, while the uninfected plants showed no hyphae.

### Aphid Performance

Anti-aphid effects of different grass-endophyte combinations were tested with the bird cherry oat aphid *Rhopalosiphum padi*, which is a severe pest species of several crop and pasture plants and often transmits the yellow dwarf virus (Whitfield et al. 2015). To test aphid performance, we added 50 adult individuals of *R. padi* aphids (Supplier: Katz Biotech AG http://www.katzbiotech.de) to each plant pot, which was then enclosed in a fine mesh bag to avoid both aphid migration between the treatments and predatory insect infestation (Fig. S2). Each treatment was replicated 12 times. To ensure equal starting conditions, all plants were cut to a height of 15 cm before adding aphids. Aphid numbers were monitored weekly for four consecutive weeks by counting for 5 min per plant pot. As 5 min were not sufficient for some treatments to cover the whole aphid population, total aphid numbers were extrapolated based on the plant area covered during 5 min of counting. Due to an even distribution of aphids, extrapolation was an appropriate measure to estimate population sizes (Fuchs et al. 2017a).

### Lacewing Larval Performance

The Common Green Lacewing (*Chrysoperla carnea*) is a representative of the order Neuroptera and is used in integrated pest management strategies. Larvae are carnivorous generalists but are widely used as predators of several aphid pest species (Atlıhan et al. 2004). Lacewing eggs were provided by Katz Biotech AG (http://www.katzbiotech.de). During their three larval stages they are efficient aphid predators with a maximum aphid consumption of approximately 100 individuals per day (Atlıhan et al. 2004). To ensure a sufficient number of aphids for feeding lacewing larvae ad libitum until pupation, five plants per treatment received 1000 individuals of *R. padi* aphids; these were reared for 6 weeks before we started feeding them to 20 newly hatched lacewing larvae per plant-endophyte combination. Lacewing larvae were kept in petri dishes in a climate cabinet with a constant temperature of 22 °C and a day/night rhythm of 16/8 hr. We used plants that had not been used for any previous experiment. Plant age, tiller number and phenology corresponded to the previously described experiment on aphid performance. Lacewing larvae were kept individually in Petri dishes to ensure proper replication and to avoid interactions between individual lacewing larvae as well as pseudo-replication. Plant parts covered with a sufficient number of aphids were added to each petri dish daily. Lacewing developmental status was recorded daily. Aphid numbers on FE+ plants did not reach sufficient population sizes to feed lacewing larvae to pupation. Consequently, data on effects of FE+ in the food chain on the development of lacewings are missing.

### Statistics

Effects of grass type on aphid population size was analyzed with *one-way* ANOVA testing aphid number depending on host plant treatment for every week independently (FE-, FEM, FE+, LE-, LE+). Since the ANOVA showed a significant difference, it was followed by *Tukey post-hoc tests*, tested for every week independently. Plant biomass on the last day had no significant effect on aphid abundances (all *p* > 0.1), analyzed as a co-factor using a *general linear model*.

Effects of grass type on lacewing larval and pupal development were analyzed with a *one-way* ANOVA for each of the larval stages and the cocoon time separately, as well as for the entire developmental time to pupation. Variances were homogeneous and data showed normal distribution. Percentage of lacewing larval mortality was analyzed with a chi-square test. Percentage of dead plant tissue was also analyzed with a chi-square test. Supplementary material contains the entire dataset from the aphid performance assay including aphid numbers (Table S1) and plant biomass with percentage of dead plant tissue (Table S2), and developmental times of lacewing larvae and pupae (Table S3).

## Results

### Aphid Performance

**Fig. 1:**
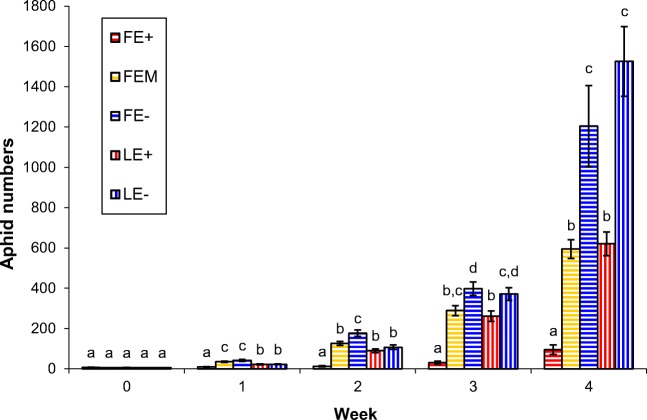
Aphid numbers (mean ± S.E) per treatment during the 5 weeks of the study. Letters indicate significant differences between treatments (*Tukey posthoc test* following *one-way* ANOVA Table 1)

One week following aphid addition to the plants (week 1), aphid population size on *Festuca arundinaceae* plants showed higher aphid numbers on FEM and FE- plants compared to FE+ plants (Table 1, Fig. 1). In the following week (week 2) aphid numbers on FE- plants were higher compared to FEM and FE+ plants, and aphid numbers on FEM plants were higher compared to FE+ (Table 1, Fig. 1). Three and 4 weeks after we started the experiment, population size on FE- plants hosted more than double the number of aphids compared to FEM plants, where we counted more than 12 times more aphids on FE- plants as compared to FE+ plants (Table 1, Fig. 1).

**Table 1:** Numbers of *R. padi*-aphids (mean ± S.E) shown for five grass treatments (*F. arundinaceae* – *E. coenophiala* common strain: FE+; *F. arundinaceae* - Moroccan-strain *E. coenophiala*: FEM, *F. arundinaceae* without endophyte: FE-; *L. perenne* – *E. festucae* var*. lolii* common toxic: LE+; *L. perenne* without endophyte: LE-); ANOVA tests were significant throughout week 1 to 4; *Tukey posthoc* statistics are indicated with letters (see also Fig. 1)

Treatment	Week 0	Week 1	Week 2	Week 3	Week 4
FE+	7.2 ± 1.2 a	9.4 ± 1.7 a	12.3 ± 3.1 a	30.9 ± 7.9 a	95.1 ± 22.8 a
FE-	6.2 ± 0.8 a	41.9 ± 4.6 c	176.4 ± 15.8 b	398.0 ± 33.4 b,c	1204.5 ± 201.9 b
FEM	5.8 ± 0.6 a	34.9 ± 3.3 c	126.3 ± 10.1 c	289.2 ± 24.6 d	594.2 ± 46.4 c
LE+	5.8 ± 0.8 a	21.8 ± 2.7 b	89.9 ± 9.6 b	261.8 ± 26.0 b	620.3 ± 59.0 b
LE-	6.0 ± 0.9 a	22.5 ± 2.1 b	107.0 ± 11.2 b	371.3 ± 31.8 c,d	1526.0 ± 173.1 c
ANOVA	*F*_4,55_ = 0.42 *p* = 0.792	*F*_4,55_ = 17.16 *p* < 0.001	*F*_4,55_ = 31.01 *p* < 0.001	*F*_4,55_ = 30.31 *p* < 0.001	*F*_4,55_ = 20.52 *p* < 0.001

Aphid population growth was slower on *Lolium perenne* plants compared to FE- and FEM, and did not differ between with and without endophyte infection until weeks 3 and 4, when populations on LE- plants showed a similar development to FE- plants, and aphid populations on LE+ plants were similar to FEM plants (Table 1, Fig. 1). By week 1 all other treatments already had higher aphid population sizes than FE+ plants (Table 1, Fig. 1).

**Fig. 2:**
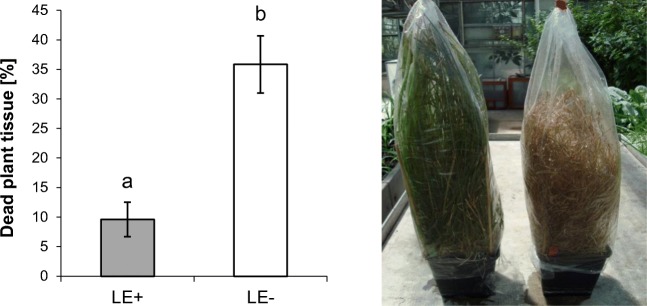
Plant damage (mean ± S.E) after 4 weeks of aphid feeding at the end of the experiment. High aphid numbers had detrimental effects on *Lolium perenne* without endophyte infection (right). Endophyte infected *L. perenne* plants were viable despite aphid infestation (left). (X^2^ = 15.12; *p* < 0.001)

Aphid population sizes were similar on endophyte-free *L. perenne* and *F. arundinaceae* plants in week 3 and week 4 (Fig. 1). The phenotype of *F. arundinacea* did not significantly differ between endophyte-infected and endophyte-free plants, with a maximum of dead tissue of approx. 20% in plants from only one FE- pot. Endophyte-free *L. perenne* plants (LE-) had on average 36% (max. 70%) dead plant tissue at the end of the study, compared to an average of 10% dead plant tissue in endophyte-infected plants (LE+) (X^2^ = 15.12; *p* < 0,001), (Fig. 2).

### Lacewing Larval Performance

**Fig. 3:**
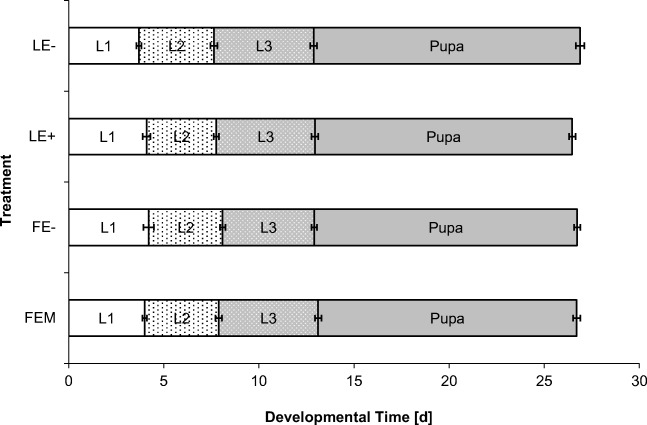
Lacewing larval developmental time after feeding on aphids reared on different grass-endophyte hosts. *One-way ANOVA* see Table 2

Feeding aphids reared on different host plants to lacewing larvae (FEM, FE-, LE+, LE-) did not result either in differences in lacewing mortality, or in the duration of the larval stages and total developmental time to pupation, analyzed with a *one-way* ANOVA (Table 2, Fig. 3). We recorded an average developmental time of 26.7 days to adult emergence.

**Table 2:** Percentage of mortality and larval developmental times (mean days ± S.E) of lacewing larvae until adult hatching

Treatment	Mortality [%]	L1 [d]	L2 [d]	L3 [d]	Pupa [d]	Total [d]
FEM	15 *n* = 20	4.0 ± 0.1 *n* = 20	3.9 ± 0.2 *n* = 19	5.2 ± 0.2 *n* = 18	13.6 ± 0.2 *n* = 17	26.7 ± 0.3 *n* = 17
FE-	20 *n* = 20	4.2 ± 0.3 *n* = 19	3.9 ± 0.1 *n* = 17	4.8 ± 0.1 *n* = 17	13.8 ± 0.2 *n* = 16	26.7 ± 0.4 *n* = 16
LE+	15 *n* = 20	4.1 ± 0.2 *n* = 20	3.7 ± 0.1 *n* = 18	5.2 ± 0.2 *n* = 17	13.5 ± 0.2 *n* = 17	26.5 ± 0.4 *n* = 17
LE-	15 *n* = 20	3.7 ± 0.1 *n* = 20	3.9 ± 0.2 *n* = 18	5.2 ± 0.2 *n* = 17	14.0 ± 0.2 *n* = 17	26.9 ± 0.3 *n* = 17
X^2^/ANOVA	X^2^ = 1.15 *p* = 0.764	*F*_3,75_ = 1.34 *p* = 0.269	*F*_3,68_ = 0.54 *p* = 0.657	*F*_3,65_ = 1.28 *p* = 0.288	*F*_3,63_ = 1.29 *p* = 0.287	*F*_3,63_ = 0.51 *p* = 0.680

## Discussion

In our experiments, aphid populations were negatively affected by endophyte infection in their host grass, independent of the plant species and its associated symbiotic fungus (i.e. *Festuca arundinaceae* with *Epichloë coenophiala*; *Lolium perenne* with *Epichloë festucae* var*. lolii*). We found that grass-endophyte chemotypes, which commonly produce high amounts of loline alkaloids (FE+ see Ball and Tapper 1999) decrease aphid fitness to a greater degree than grass-endophytes which are known to produce less or no loline alkaloids (FEM, LE+ see Siegel et al. 1990; Ball and Tapper 1999). In addition, our results demonstrate the impact of aphids on endophyte-free perennial ryegrass; an enormous decrease in plant quality resulting in high proportions of dead plant material.

Studies on the effectiveness of grass-endophytes against herbivores have varied widely, depending not only on biotic and abiotic conditions but also on the specific alkaloid composition produced by each grass-endophyte association (Hunt and Newman 2005; Bultman et al. 2006; Fuchs et al. 2017c). Our study demonstrated that chemotypes associated with high loline concentrations are most effective against *R. padi* aphids, as evidenced by the result that it was not even possible to rear sufficient individuals under this condition for our lacewing performance experiment. Observed low aphid population sizes on *F. arundinaceae* infected with *E. coenophiala* common strain illustrated the effectiveness of grass endophytes in aphid control. Grass-endophyte chemotypes producing low amounts of lolines (FEM) or lacking these compounds (LE+) hosted lower aphid numbers compared to uninfected grass but significantly more aphids compared to FE+ plants, even after only 1 week, and this persisted to the end of the study. It is important to state, that we were not able to measure alkaloid concentrations in the plants as well as aphids due to technical limitations at the time of the study. However, as we used chemically well characterized grass-endophyte combinations, we will rely on the reported alkaloid compositions to draw correlations. We demonstrated the effectiveness of grass-endophytes in aphid control, which correlates well with the assumed loline concentrations in the tested plant-endophyte associations (Wilkinson et al. 2000; Hunt and Newman 2005; Bultman et al. 2006). The lack of genes responsible for the biosynthesis of ergovaline, N-formylloline and N-acetylloline in the Moroccan strain of *E. coenophiala* might also contribute to reduced efficiency against herbivores (Ball and Tapper 1999). Planting selected grass-endophyte strains surrounding crop areas may be a first step in decreasing rapid aphid outbreaks, due to the toxic effect of lolines on aphids. Additionally, grass-endophytes alter the emission of volatile organic compounds (VOCs) of their host grass, which potentially repel aphids away from the plant or may even attract predatory insects (Li et al. 2014; Turlings and Erb 2018; Fuchs and Krauss 2019).

Despite their excellent properties for pest control, endophytes can harm beneficial insects by cascading toxins through the food chain from aphids to higher trophic levels (Fuchs et al. 2013); this was shown for the predatory insect the seven-spotted ladybird *Coccinella septempunctata*, the aphid parasitoid *Aphidius ervi*, and even a secondary parasitoid *Asaphes vulgaris* (de Sassi et al. 2006; Härri et al. 2008a, 2008b). It has been demonstrated, that especially peramine and lolitrem B cascade-up the food chain, which are likely responsible for observed fitness disadvantages found at higher trophic levels (Fuchs et al. 2013). Most of those studies were conducted with loline-free endophyte-infected *L. perenne*, but similar effects were recorded for loline-containing *F. arundinaceae* on an aphid and lepidopteran parasitoid species (Bultman et al. 1997, 2012).

Lacewings are one of the major natural aphid control agents in agricultural systems and have demonstrated resistance to certain challenging biological and chemical pesticides, which makes them a preferred and reliable biological pest control agent (Pree et al. 1989; Tian et al. 2013). Lacewings were in our experimental setup not susceptible to influences of endophyte infection in their food chain, suggesting that endophyte-mediated bottom-up and lacewing-mediated top-down control of aphid populations may be able to coexist without directly affecting each other. Furthermore, bottom-up cascades of alkaloid concentrations may have been low due to plant age (Fuchs et al. 2017c), which may be responsible for missing effects on lacewings. However, younger plants used in a previous study did exhibit cascading negative effects on the fitness of ladybirds as the third trophic level (de Sassi et al. 2006). In order to confirm, whether *C. carnea* larvae are tolerant to alkaloids, follow-up studies need to incorporate high performance liquid chromatography and mass spectrometry experiments to measure alkaloid levels in aphids and lacewings.

Lolines are classified as pyrrolizidine alkaloids, widespread plant-derived insecticides shaping multi-trophic interactions (Hartmann 1999). Several herbivores have developed mechanisms to cope with plant-produced pyrrolizidine alkaloids and are able to sequester these compounds, thereby improving their own chemical defenses (Trigo 2011). However, in most cases only specialist herbivores show adaptations to these alkaloids, whereas at higher trophic levels variable effects have been observed, differing among plant and insect species (Trigo 2011).

Predatory insects may further be deterred by pyrrolizidine-containing prey, as shown for lacewings feeding on certain moth eggs (Bezzerides et al. 2004). Future studies should focus on effects of endophyte-derived lolines at higher trophic levels. Furthermore, other beneficial insect species should be tested for their susceptibility to endophyte-derived alkaloids; syrphid fly larvae, for example, are efficient aphid predators (Smith et al. 2008) and were observed in higher numbers on endophyte-infected *Lolium perenne* during a common-garden study (Fuchs and Krauss 2019). Our results indicate that bottom-up and top-down control of crop pests have the potential to be applied synergistically. Future studies are needed to verify our results under field conditions, where the preference of aphids and their predators to different grass-endophyte strains should be tested.

Our results suggest that *F. arundinacea* infected with *E. coenophiala* common strain is highly effective against aphid pests and should be considered as an agent capable of reducing aphid populations in grass pastures. The endophyte-infected grass may further be utilized by planting to surround crop fields, preventing aphid outbreaks and decreasing the distribution of vector-borne diseases such as the yellow-dwarf virus, which is responsible for tremendous yield losses in cereal crops (Burnett 1990). To prevent unwanted effects on livestock, the Moroccan strain, lacking vertebrate toxic alkaloids, is a “livestock-safe” alternative to the common strain, but produces low levels of lolines and is not as effective against aphids as the common strain. Implementing both bottom-up and top-down strategies in sustainable and biological pest control is a promising strategy for effective biocontrol to replace chemical pesticides. To maximize the effectiveness of merging different approaches in biological pest control, a deeper understanding is needed of how they affect each other both directly and indirectly.

## Electronic supplementary material

ESM 1 (DOCX 1050 kb)
