# Supplementary material for: Contrasting Effects of Grass - Endophyte Chemotypes on a Tri-Trophic Cascade
Source: J Chem Ecol. 2020 Mar 3;46(4):422–9. doi: 10.1007/s10886-020-01163-9 (PMC7205845; doi:10.1007/s10886-020-01163-9)
Supplement: Supplementary file 1 — (DOCX 1050 kb) [file 10886_2020_1163_MOESM1_ESM.docx]

Supplementary material

Fig S1


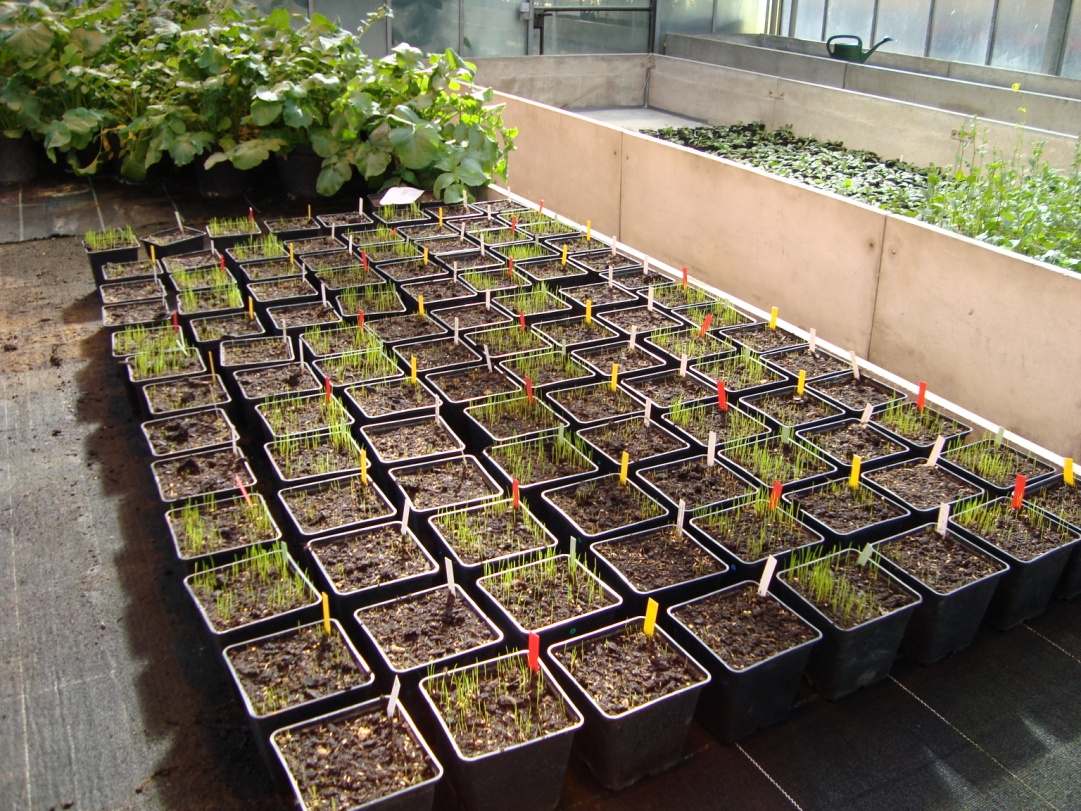


Fig S1: Randomly arranged pots, each sown with 200 grass seeds

Fig S2


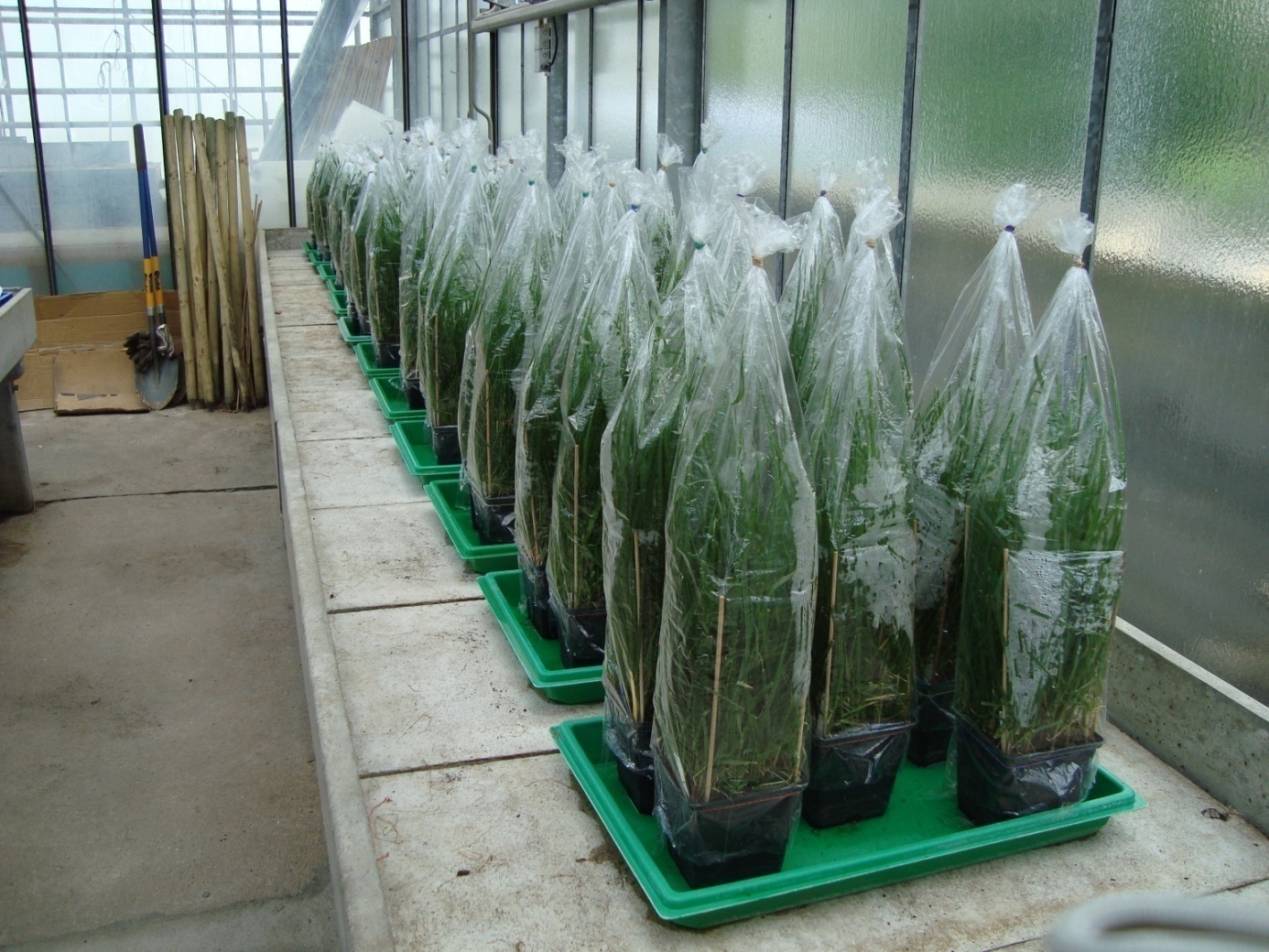


Fig S2: Plant setup with meshbags allowing air circulation but preventing insect movement between the treatments. Each of the five treatments was randomly placed in each of the 12 trays.

Table S1: Aphid numbers per pot during the entire study

| Nr. | grass | week0 | week1 | wek2 | week3 | week4 |
| --- | --- | --- | --- | --- | --- | --- |
| 1 | FE+ | 4 | 5 | 0 | 3 | 31 |
| 2 | FE- | 3 | 36 | 165 | 507 | 1368 |
| 3 | FEM | 5 | 23 | 102 | 221 | 780 |
| 4 | FE+ | 3 | 5 | 8 | 33 | 88 |
| 5 | FE- | 7 | 35 | 176 | 363 | 783 |
| 6 | LE+ | 3 | 15 | 73 | 218 | 582 |
| 7 | FE- | 2 | 44 | 153 | 231 | 447 |
| 8 | LE- | 5 | 26 | 92 | 318 | 1236 |
| 9 | LE+ | 5 | 24 | 70 | 308 | 690 |
| 10 | FE- | 7 | 46 | 181 | 490 | 2500 |
| 11 | FEM | 5 | 21 | 102 | 149 | 246 |
| 12 | LE- | 5 | 26 | 76 | 304 | 832 |
| 13 | FE- | 6 | 51 | 161 | 426 | 1600 |
| 14 | LE+ | 7 | 8 | 48 | 136 | 334 |
| 15 | LE+ | 9 | 26 | 112 | 458 | 900 |
| 16 | FE+ | 3 | 17 | 18 | 24 | 71 |
| 17 | LE- | 9 | 22 | 58 | 351 | 1000 |
| 18 | LE+ | 9 | 20 | 80 | 246 | 611 |
| 19 | FEM | 4 | 24 | 65 | 185 | 376 |
| 20 | FE+ | 6 | 5 | 5 | 31 | 153 |
| 21 | FE+ | 9 | 22 | 32 | 93 | 192 |
| 22 | LE- | 0 | 15 | 143 | 445 | 2400 |
| 23 | FEM | 6 | 35 | 138 | 313 | 565 |
| 24 | FE+ | 6 | 6 | 9 | 5 | 27 |
| 25 | LE- | 6 | 12 | 84 | 299 | 1400 |
| 26 | FEM | 6 | 32 | 112 | 292 | 459 |
| 27 | LE+ | 5 | 20 | 111 | 247 | 412 |
| 28 | LE- | 6 | 21 | 42 | 143 | 444 |
| 29 | FE+ | 5 | 6 | 5 | 27 | 41 |
| 30 | FEM | 5 | 39 | 108 | 305 | 621 |
| 31 | LE+ | 8 | 26 | 76 | 162 | 326 |
| 32 | FEM | 9 | 42 | 173 | 424 | 730 |
| 33 | LE+ | 5 | 29 | 115 | 341 | 710 |
| 34 | FEM | 5 | 55 | 185 | 364 | 742 |
| 35 | FE+ | 4 | 3 | 0 | 7 | 28 |
| 36 | FE+ | 9 | 6 | 5 | 9 | 18 |
| 37 | FEM | 2 | 29 | 146 | 244 | 586 |
| 38 | LE+ | 1 | 5 | 28 | 170 | 578 |
| 39 | LE- | 10 | 14 | 121 | 405 | 1600 |
| 40 | LE- | 9 | 22 | 156 | 521 | 1400 |
| 41 | LE- | 3 | 27 | 110 | 356 | 1800 |
| 42 | FE- | 7 | 25 | 146 | 385 | 618 |
| 43 | FE- | 10 | 36 | 103 | 186 | 456 |
| 44 | FEM | 7 | 53 | 132 | 312 | 632 |
| 45 | LE- | 7 | 24 | 98 | 310 | 2000 |
| 46 | LE+ | 9 | 18 | 97 | 234 | 590 |
| 47 | LE+ | 3 | 35 | 133 | 307 | 710 |
| 48 | FE+ | 17 | 13 | 15 | 22 | 81 |
| 49 | FE+ | 10 | 10 | 28 | 69 | 272 |
| 50 | FE- | 5 | 34 | 142 | 457 | 1032 |
| 51 | LE- | 3 | 22 | 163 | 473 | 1800 |
| 52 | FE- | 11 | 86 | 322 | 530 | 1200 |
| 53 | FE- | 8 | 42 | 232 | 530 | 2000 |
| 54 | FEM | 9 | 25 | 97 | 246 | 684 |
| 55 | LE- | 9 | 39 | 141 | 530 | 2400 |
| 56 | FE+ | 10 | 15 | 23 | 48 | 139 |
| 57 | FE- | 5 | 26 | 171 | 391 | 2000 |
| 58 | FE- | 3 | 42 | 165 | 280 | 450 |
| 59 | LE+ | 6 | 35 | 136 | 314 | 1000 |
| 60 | FEM | 7 | 41 | 155 | 415 | 709 |

Table S2: Plant biomass and percentage of dead plant tissue at the end of the aphid population growth experiment

| Nr. | grass | biomass g | Dead plant tissue [%] |
| --- | --- | --- | --- |
| 1 | FE+ | 20.7 | 0 |
| 2 | FE- | 17.9 | 0 |
| 3 | FEM | 19.7 | 0 |
| 4 | FE+ | 20.8 | 0 |
| 5 | FE- | 19.4 | 0 |
| 6 | LE+ | 15.5 | 30 |
| 7 | FE- | 19 | 0 |
| 8 | LE- | 14.9 | 30 |
| 9 | LE+ | 15.3 | 0 |
| 10 | FE- | 19.2 | 0 |
| 11 | FEM | 16.5 | 0 |
| 12 | LE- | 16 | 30 |
| 13 | FE- | 16.3 | 20 |
| 14 | LE+ | 19.5 | 0 |
| 15 | LE+ | 13.6 | 20 |
| 16 | FE+ | 18.9 | 0 |
| 17 | LE- | 14.6 | 30 |
| 18 | LE+ | 11.8 | 10 |
| 19 | FEM | 19.6 | 0 |
| 20 | FE+ | 17.1 | 0 |
| 21 | FE+ | 20.2 | 0 |
| 22 | LE- | 14.8 | 50 |
| 23 | FEM | 22.4 | 0 |
| 24 | FE+ | 19.6 | 0 |
| 25 | LE- | 15.4 | 30 |
| 26 | FEM | 22.7 | 0 |
| 27 | LE+ | 18.9 | 0 |
| 28 | LE- | 14.8 | 0 |
| 29 | FE+ | 22.3 | 0 |
| 30 | FEM | 20.1 | 0 |
| 31 | LE+ | 16.9 | 0 |
| 32 | FEM | 20.2 | 0 |
| 33 | LE+ | 16.8 | 0 |
| 34 | FEM | 24 | 0 |
| 35 | FE+ | 30.7 | 0 |
| 36 | FE+ | 24.8 | 0 |
| 37 | FEM | 23 | 0 |
| 38 | LE+ | 16.4 | 20 |
| 39 | LE- | 15.4 | 30 |
| 40 | LE- | 15.3 | 30 |
| 41 | LE- | 14.8 | 40 |
| 42 | FE- | 20.6 | 0 |
| 43 | FE- | 18.2 | 0 |
| 44 | FEM | 25.6 | 0 |
| 45 | LE- | 19.9 | 50 |
| 46 | LE+ | 17.1 | 10 |
| 47 | LE+ | 19.9 | 10 |
| 48 | FE+ | 21.2 | 0 |
| 49 | FE+ | 21.2 | 0 |
| 50 | FE- | 21.3 | 0 |
| 51 | LE- | 15.5 | 40 |
| 52 | FE- | 19.7 | 0 |
| 53 | FE- | 17.7 | 0 |
| 54 | FEM | 24.7 | 0 |
| 55 | LE- | 17.1 | 70 |
| 56 | FE+ | 26.7 | 0 |
| 57 | FE- | 22.5 | 0 |
| 58 | FE- | 21.2 | 0 |
| 59 | LE+ | 18.7 | 15 |
| 60 | FEM | 25.7 | 0 |

Table S3: Mortality and developmental time for each lacewing larvae

| Nr. | grass | L1 [d] | L2 [d] | L3 [d] | Kokon [d] | died |
| --- | --- | --- | --- | --- | --- | --- |
| 1 | LE+ | 4 | 3 | 5 | 13 | n |
| 2 | FEM | 4 | 4 | 4 | 14 | n |
| 3 | FE- | 3 | 4 | 5 | 14 | n |
| 4 | LE- | 4 | 4 | 5 | 14 | n |
| 5 | LE+ | 3 | 3 | 5 | 13 | n |
| 6 | FEM | 4 | 3 | 5 | 13 | n |
| 7 | FE- | 4 | 4 | 4 | 13 | n |
| 8 | LE- | 3 | 4 | 5 | 13 | n |
| 9 | FE- | 3 | 4 | 5 | NA | y |
| 10 | FEM | 4 | 4 | 6 | 15 | n |
| 11 | LE+ | 4 | 4 | 4 | 14 | n |
| 12 | LE- | 4 | 4 | 5 | 14 | n |
| 13 | FE- | 4 | 4 | 6 | 14 | n |
| 14 | FEM | 3 | 4 | 6 | 14 | n |
| 15 | LE- | 4 | 4 | 5 | 14 | n |
| 16 | LE+ | 5 | 3 | 6 | 15 | n |
| 17 | FE- | 4 | 5 | 5 | 14 | n |
| 18 | FEM | 4 | 4 | 4 | 13 | n |
| 19 | LE- | 4 | 4 | 5 | 14 | n |
| 20 | LE+ | 5 | 4 | 4 | 13 | n |
| 21 | FEM | 4 | 4 | 5 | 13 | n |
| 22 | FE- | 5 | 3 | 6 | 15 | n |
| 23 | LE+ | 5 | 3 | 5 | 13 | n |
| 24 | LE- | 4 | 4 | 5 | 14 | n |
| 25 | FE- | 5 | 3 | 4 | 13 | n |
| 26 | FEM | 4 | 4 | 5 | 14 | n |
| 27 | LE- | 4 | 4 | 4 | 14 | n |
| 28 | LE+ | 5 | 3 | 5 | 12 | n |
| 29 | FE- | 3 | 4 | 5 | 13 | n |
| 30 | FEM | 4 | 4 | 5 | NA | y |
| 31 | LE+ | 3 | 4 | 6 | 14 | n |
| 32 | LE- | 3 | 4 | 6 | 14 | n |
| 33 | FE- | 4 | 4 | 5 | 14 | n |
| 34 | FEM | 4 | 4 | 5 | 13 | n |
| 35 | LE- | 4 | 3 | 6 | 13 | n |
| 36 | LE+ | 3 | 4 | 5 | 13 | n |
| 37 | FE- | 3 | 4 | 5 | 14 | n |
| 38 | FEM | 4 | 3 | 6 | 12 | n |
| 39 | LE- | 3 | 6 | 4 | 13 | n |
| 40 | LE+ | 3 | NA | NA | NA | y |
| 41 | FE- | 3 | 5 | 4 | 14 | n |
| 42 | FEM | 3 | 5 | 6 | 15 | n |
| 43 | LE- | 3 | 3 | 6 | 13 | n |
| 44 | LE+ | 5 | 4 | NA | NA | y |
| 45 | FE- | 4 | 3 | 5 | 13 | n |
| 46 | FEM | 5 | 4 | 6 | 13 | n |
| 47 | LE- | 4 | 3 | 6 | 14 | n |
| 48 | LE+ | 4 | 4 | 5 | 14 | n |
| 49 | FE- | 8 | 4 | 4 | 14 | n |
| 50 | FEM | 5 | 4 | 4 | 14 | n |
| 51 | LE- | 4 | 3 | 5 | 14 | n |
| 52 | LE+ | 5 | 3 | 5 | 13 | n |
| 53 | FE- | 5 | NA | NA | NA | y |
| 54 | FEM | 5 | 3 | 5 | 13 | n |
| 55 | LE- | 4 | 6 | NA | NA | y |
| 56 | LE+ | 3 | 5 | 5 | 14 | n |
| 57 | FE- | NA | NA | NA | NA | y |
| 58 | FEM | 4 | NA | NA | NA | y |
| 59 | LE- | 3 | NA | NA | NA | y |
| 60 | LE+ | 3 | NA | NA | NA | y |
| 61 | FE- | 5 | 4 | 5 | 14 | n |
| 62 | FEM | 4 | 6 | NA | NA | y |
| 63 | LE- | 4 | 4 | 5 | 15 | n |
| 64 | LE+ | 5 | 4 | 6 | 14 | n |
| 65 | FE- | 5 | 4 | 5 | 14 | n |
| 66 | FEM | 4 | 3 | 5 | 14 | n |
| 67 | LE- | 5 | 3 | 7 | 14 | n |
| 68 | LE+ | 3 | 4 | 5 | 14 | n |
| 69 | FE- | 4 | NA | NA | NA | y |
| 70 | FEM | 3 | 4 | 6 | 14 | n |
| 71 | LE- | 3 | NA | NA | NA | y |
| 72 | LE+ | 5 | 4 | 7 | 13 | n |
| 73 | FE- | 4 | 3 | 4 | 13 | n |
| 74 | FEM | 4 | 3 | 6 | 14 | n |
| 75 | LE- | 3 | 4 | 5 | 17 | n |
| 76 | LE+ | 4 | 3 | 5 | 14 | n |
| 77 | FE- | 4 | 4 | 5 | 15 | n |
| 78 | FEM | 4 | 4 | 5 | 13 | n |
| 79 | LE- | 4 | 4 | 5 | 14 | n |
| 80 | LE+ | 5 | 4 | 5 | 14 | n |
